# Neuromuscular and Neuroendocrinological Features Associated With *ZC4H2*-Related Arthrogryposis Multiplex Congenita in a Sicilian Family: A Case Report

**DOI:** 10.3389/fneur.2021.704747

**Published:** 2021-07-12

**Authors:** Gianluca Piccolo, Giuseppe d'Annunzio, Elisabetta Amadori, Antonella Riva, Paola Borgia, Domenico Tortora, Mohamad Maghnie, Carlo Minetti, Eloisa Gitto, Michele Iacomino, Simona Baldassari, Chiara Fiorillo, Federico Zara, Pasquale Striano, Vincenzo Salpietro

**Affiliations:** ^1^Pediatric Neurology and Muscular Diseases Unit, IRCCS Istituto Giannina Gaslini, Genoa, Italy; ^2^Department of Pediatrics, IRCCS Istituto Giannina Gaslini, University of Genoa, Genoa, Italy; ^3^Pediatric Clinic and Endocrinology, Regional Center for Pediatric Diabetes, IRCCS Istituto Giannina Gaslini, Genoa, Italy; ^4^Department of Neurosciences, Rehabilitation, Ophthalmology, Genetics, Maternal and Child Health (DINOGMI), University of Genoa, Genoa, Italy; ^5^Unit of Medical Genetics, IRCCS Istituto Giannina Gaslini, Genoa, Italy; ^6^Neuroradiology Unit, IRCCS Istituto Giannina Gaslini, Genoa, Italy; ^7^Department of Human Pathology of the Adult and Developmental Age, “Gaetano Barresi” University of Messina, Messina, Italy

**Keywords:** arthrogryposis, *ZC4H2*, neurodevelopmental disorders, Wieacker-Wolff syndrome, exome sequencing, recurrent hypoglycemic events, neuromuscular junction, case report

## Abstract

Wieacker-Wolff syndrome (WWS) is an X-linked Arthrogryposis Multiplex Congenita (AMC) disorder associated with broad neurodevelopmental impairment. The genetic basis of WWS lies in hemizygous pathogenic variants in *ZC4H2*, encoding a C4H2 type zinc-finger nuclear factor abundantly expressed in the developing human brain. The main clinical features described in WWS families carrying *ZC4H2* pathogenic variants encompass having a short stature, microcephaly, birth respiratory distress, arthrogryposis, hypotonia, distal muscle weakness, and broad neurodevelopmental delay. We hereby report a Sicilian family with a boy clinically diagnosed with WWS and genetically investigated with exome sequencing (ES), leading to the identification of a c.593G>A (p. R198Q) hemizygous pathogenic variant in the *ZC4H2* gene. During the first year of life, the onset of central hypoadrenalism led to recurrent hypoglycemic events, which likely contributed to seizure susceptibility. Also, muscle biopsy studies confirmed a pathology of the muscle tissue and revealed peculiar abnormalities of the neuromuscular junction. In conclusion, we expand the phenotypic spectrum of the WWS-related neurodevelopmental disorders and discuss the role of *ZC4H2* in the context of the potential neuroendocrinological and neuromuscular features associated with this condition.

## Introduction

Arthrogryposis Multiplex Congenita (AMC) is a rare neuromuscular condition with an incidence of about 1 in 3,000–5,000 live births (1), characterized by non-progressive congenital contractures involving at least two joints (2). The underlying genetic causes and the subtle disease mechanisms associated with AMC are heterogeneous and not yet completely understood (3). In recent years, next generation sequencing technologies, including exome and genome studies, revealed an increased complexity underlying infantile-onset neuromuscular disorders associated with neurodevelopmental impairment (4–6). Many novel genes have been identified with consequent benefits in terms of refining clinical phenotypes, valuable prognostic information, and targeted therapies (7–9).

Pathogenic variants in *ZC4H2* cause an ultra-rare peculiar X-linked AMC disorder described as Wieacker-Wolff syndrome (WWS; MIM# 314580), also defined as Miles-Carpenter syndrome (MCS) (10). WWS is also considered an X-linked intellectual disability/neurodevelopmental gene (XLID), and the genotype-phenotype spectrum associated with *ZC4H2* variants is limited due to the few cases reported in the literature to date (11).

In this study, we report the case of an Italian child affected by WWS. The boy presented a combination of endocrinological comorbidities, including congenital hypothyroidism and recurrent hypoglycemic events, that led to the diagnosis of central hypoadrenalism in the context of classical WWS. We also revise the *ZC4H2*-related molecular functions and clinical phenotypes, speculating on the possible pathophysiology of the complex endocrinological and neuromuscular features observed in our patient.

## Patient Presentation

The boy was born from unrelated healthy parents of Italian descent and presented with hypotonia, arthrogryposis (mainly distal), and severe respiratory distress since birth. Family history was unremarkable for arthrogryposis and neurodevelopmental disorders. Pregnancy was complicated by the detection of oligohydramnios and pathological prenatal reduction of the fetal heart rate. Thus, an urgent cesarean section was performed at the gestational age of 34 + 6 weeks. At birth, the APGAR score was 5 at 60” and 9 at 5'. Growth parameters recorded on the first day of life were all within normal limits (weight: 2,130 g; length: 47 cm; occipitofrontal circumference: 32 cm).

The patient required endotracheal intubation and stayed at the pediatric intensive care unit for the first 6 weeks of life due to respiratory distress syndrome and necrotizing enterocolitis. He presented with bilateral severe hip dysplasia, mild mitral insufficiency, *pectus carinatum*, arthrogryposis of hands, knees, and feet (congenital vertical talus), and bilateral cryptorchidism. Neonatal screening for inborn errors of metabolism revealed central hypothyroidism, while both visual evoked potentials and brainstem auditory evoked potentials were abnormal.

Two cardio-respiratory arrests occurred in the first week of life, requiring cardiopulmonary resuscitation. At that time, Holter-ECG was performed, and prolonged (repeated) heart pauses associated with bradycardia were recorded. At the age of 1 month, the boy showed tonic-clonic seizures requiring treatment with antiepileptic drugs (i.e., phenobarbital and levetiracetam). His EEG showed abundant and polymorphic occipital delta waves, while the head CT scan did show ventriculomegaly and bilateral frontal-temporal cerebral atrophy. The metabolic work-up (i.e., urinary organic acids, blood amino acids, and sialotransferrine profile) was reported as normal. Suspecting the co-existence of gastro-esophageal reflux, a transit X-ray study was then performed; the patient presented with cardiac incontinence (gaping) associated with distal esophageal stenosis, requiring urgent Nissen fundoplication and percutaneous endoscopic gastrostomy. After being discharged, no feeding difficulties were reported, though the boy presented inadequate growth parameters (weight constantly below the third centile) and severe neurodevelopmental delay. At the age of 9 months, he presented with poor general motility, lack of eye contact, horizontal nystagmus, divergent strabismus, and delay of motor milestones. Distinctive facial features included bi-temporal narrowing, a highly arched palate, mandibular hypoplasia, and large posteriorly rotated ears. Moreover, short neck, *pterigium colli*, nipples hypoplasia, and *pectus carinatum* were also present. Severe motor delay with global hypotonia and muscle hypotrophy was also evident. At the age of 12 months, he could not get to the sitting position on his own nor remain seated. Neuromotor evaluation, besides multiple joint retractions (knees, ankle, and feet) suggestive of arthrogryposis, also revealed myopathic face with strabismus, absent tendon reflexes, and rigid spine. Nerve conduction study documented only reduced amplitude of the tibialis motor potential. Sensory nerve velocities and potentials were normal. Electromyography study excluded the presence of denervation or myopathic damage.

**Figure 1:**
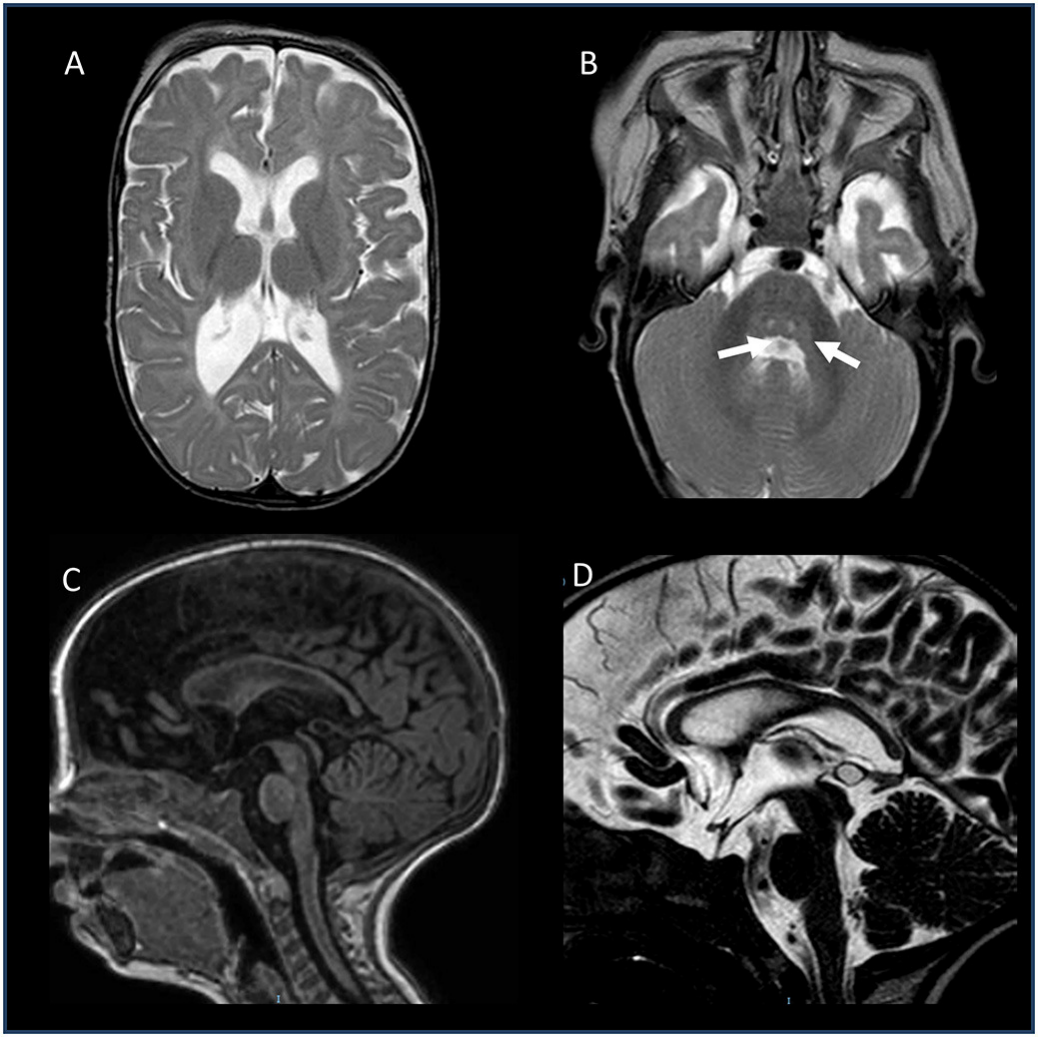
Brain MRI of the patient, performed at the age of 8 months. **(A,B)** Axial T2-weighted images: enlarged posterior horn of lateral ventricles with periventricular white matter thinning **(A)** and central tegmental tracts hyperintensity (white arrows). **(C)** Sagittal T1-weighted image and **(D)** Sagittal T2-DRIVE: Thinning of the corpus callosum and mild pons hypoplasia. No abnormalities recorded in the hypothalamic-pituitary axis.

**Figure 2:**
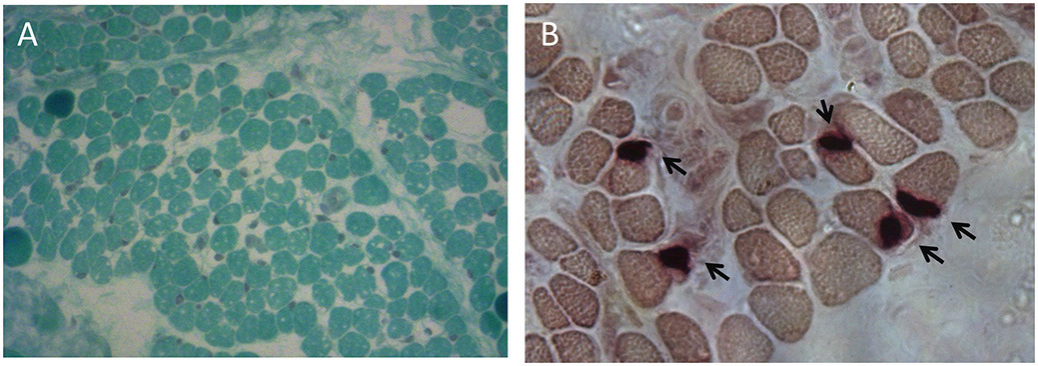
Muscle biopsy performed at the age of 9 months. **(A)** Modified Gomori Trichrome staining showing round and small muscle fibers separated by increased connective tissue and containing several cytoplasmic vacuoles. **(B)** Non-specific esterase (NSE) staining displaying swelled NMJs.

To further investigate possible central nervous system abnormalities, when he was 8 months old, the boy underwent a brain Magnetic Resonance Imaging (MRI), which showed typical signs of hypoxic-ischemic encephalopathy, in association with a diffuse white matter myelination delay—more pronounced in both optic radiations—and pons hypoplasia (Figure 1). A muscle biopsy, performed at the age of 9 months, revealed signs of muscle damage including round and small fibers and the presence of cytoplasmic vacuoles. In addition, using specific staining, we also detected enlargement and abnormality on the shape of the neuromuscular junctions (NMJ; Figure 2).

Soon after his first birthday, he was admitted to the Emergency Department for a prolonged seizure lasting for ~10 min, and not resolving after endorectal diazepam administration. During admission, severe hypoglycemia (<1.1 mmol/L) and low plasmatic cortisol levels were detected. Hence, the boy was diagnosed with central hypoadrenalism, and replacement therapy (hydrocortisone) was started. A Growth Hormone Releasing Hormone (GHRH)-arginine stimulation test was then performed: growth hormone levels resulted in the normal range of response.

Genetic studies, including karyotype, array-Comparative Genome Hybridization (CGH), Sanger sequencing of the *MAGEL2* gene (due to a clinical suspect of Schaaf-Yang disease), and a gene panel including *n* = 52 genes associated with neurodevelopmental disorders and epileptic encephalopathies, were all reported as normal. To investigate the molecular etiology of the phenotype in the affected individual, we then performed exome sequencing (ES) of the proband and his parents (12, 13). This led to the identification of a pathogenic hemizygous variant in *ZC4H2* [NM_018684.4: c.593G>A (p. R198Q)], predicted as deleterious by several *in-silico* tools, including CADD, SIFT, PolyPhen, and MutationTaster. The variant is also regarded as pathogenic in ClinVar (VCV000050981.1; https://www.ncbi.nlm.nih.gov/clinvar, last accessed May 31, 2021).

## Discussion

Genetic neuromuscular and developmental disorders with onset in the first year of life include a variety of monogenic conditions, including AMC, with expanding clinical differential diagnosis, genetic heterogeneity, and associated disease mechanisms (14–17).

The *ZC4H2* gene has been implicated in the pathogenesis of AMC, namely WWS (10). It is located at Xq11.2 and encodes a C4H2 type zinc-finger nuclear factor, composed by both a C-terminal zinc finger domain and a coiled-coil domain. Given the high expression of *ZC4H2* within the antenatal and early postnatal brain, an important role of this gene in the development and function of the central nervous system has been postulated (18).

Yet, the main clinical findings reported in families with *ZC4H2* mutations include having a short stature and microcephaly, respiratory distress at birth, motor developmental delay with associated muscle weakness and hypotonia, distal contractures, hip dislocation, club foot, short neck, and broad neurodevelopmental impairment affecting multiple domains (i.e., motor, language, and cognition) (19). The different combinations of these signs and symptoms, being the consequence of various allelic mutations in *ZC4H2*, have also been referred to as *ZC4H2*-Associated Rare Disorders (ZARD) (20).

Life expectancy in patients affected by ZARD is still not clear. Hirata et al. (18) described a German family of five affected individuals carrying the p.R198Q pathogenic variant, all characterized by intellectual disability, spasticity, and equinovarus feet; three presented with neonatal respiratory distress; they were reported to have expired between 0.8 and 8 years (18). More recently, Frints et al. (20) reported two additional patients carrying the same p.R198Q variant, both presenting with recurrent prolonged episodes of bradycardia and apnea, requiring cardiopulmonary resuscitation, and in one case, pacemaker implantation. One of these individuals died at the age of 8 months. (20). Our patient is currently 3 years old, fed via a gastrostomy, and under hydrocortisone and thyroid hormone therapy. Seeing the reported episodes of bradycardia and cardiac arrest, a close cardiological follow-up should be assessed and pacemaker implantation should be considered.

On the MRI findings, notably, in our patient, they were consistent with those described both in the case by Zanzottera et al. (21)—thin brainstem and corpus callosum—and by Wang et al. (22)—slight ventricular enlargement and diffuse cerebral atrophy.

In the case by Zanzottera et al. (21)—a female individual affected with WWS associated with progressive damages of muscles and peripheral nerves—electromyography (EMG) performed at 13 years of age showed evident neurogenic signs in the deltoid muscle (increased amplitude and duration of motor unit) as well as moderate myopathic anomalies at distal muscles of the lower limbs. Consistent with the EMG features, muscle biopsy of this case revealed type II hypotrophy. In five affected individuals, Hirata et al. showed a reduction of the muscle fiber size only during the neonatal period, and then, these features normalized around 1 year of age (18). Conversely, our patient's muscle biopsy at the age of 9 months revealed moderate muscle damage with associated fiber hypotrophy, vacuoles, and enlargement of the NMJs.

Histopathological abnormalities of the NMJs have been described in forms of congenital myasthenic syndrome (23), and these syndromes can clinically manifest with arthrogryposis or fetal akinesia syndrome (24) as a consequence of the reduced movement during pregnancy. Thus, we suggest that defects of the NMJ, such as the ones we have observed in our patient, can potentially contribute to the motor and arthrogryposis phenotype of WWS and similar conditions. Interestingly, morpholino-mediated knockdown of *ZC4H2* in zebrafish resulted in reduced numbers of neuromuscular endplates and endplates disorganization (18). Further studies on *ZC4H2* localization in humans are warranted to support this hypothesis.

Importantly, despite the fact that WWS is an X-linked recessive disorder, females carrying heterozygous *ZC4H2* mutations may display a mild intellectual disability phenotype as well as subtle distal contractures and sometimes clubfoot (21, 25). A recent study by Wang et al. (22) demonstrated that mechanisms underlying X-chromosome inactivation (XCI) in females are not necessarily implicated in the disease phenotype. In this regard, XCI seems to be correlated with a milder phenotype in female carriers (22).

At present, it is well known that both RNF220 and its co-factor *ZC4H2* are required for multiple neural developmental processes, including spinal cord patterning, cerebellum embryogenesis and function, and activity of the *locus coeruleus* (26). A study by Ma et al. using *Zc4h2* knockdown mice models revealed peculiar alterations in the V2a and V2b hindbrain and spinal cord interneurons, potentially implicating such features in the neuronal (and thus neurodevelopmental) phenotypes associated with WWS (27). Interestingly, *ZC4H2* forms a complex with RNF200 (a ubiquitin E3 ligase) and is required for its stability. Kim et al. suggested that the RNF200/*ZC4H2* complex could modulate transcription factors such as Dbx1, Dbx2, and Nkx2.2, thus being directly involved in the transcriptional network that regulates neural patterning (28). More recently, Song et al. demonstrated that in mice brain, the RNF200/*ZC4H2* complex allows the full transcriptional activity of Phox2a and Phox2b through monoubiquitylation, thus playing a pivotal role in the development of noradrenergic neurons in the *locus coeruleus* (29).

Notably, a rare occurrence of hypoglycemic events is reported in *ZC4H2* mutation carriers. In a previous study, hypoglycemia was described as a potential metabolic feature in patients carrying the p.R198Q and p.R213W pathogenic hemizygous variants (30). Kondo et al. (30) also described two siblings carrying the missense variant p.K209N presenting with recurrent post-prandial hypoglycemia (due to an increased insulin secretion). In this case, the authors speculated on the possible involvement of the bone morphogenic protein (BMP) signaling pathway, the *ZC4H2* protein being involved in the regulation of SMAD family proteins, which are involved in the BMP signaling (27, 30). Intriguingly, in another study by Ma et al., a group of *ZC4H2* substitutional mutations (V63L, A200V, P201S, R198Q, and R213W) was tested on *Xenopus* mutants: they all failed to stabilize SMAD *in vivo* except for R198Q (31).

The affected individual reported in our study presented with persistent severe hypoglycemia due to a confirmed diagnosis of central hypoadrenalism. Central hypoadrenalism may lead to a reduced secretion of cortisol and subsequent reduction of hepatic gluconeogenesis, glucagon secretion, and increased activity of insulin receptors (32). To note, defects in adrenal function have been identified in several neurological conditions and genetic syndromes and may sometimes represent a peculiar trait of complex clinical phenotypes (33, 34). Thus, we suggest to carefully consider the contribution of central hypoadrenalism as a potential feature within the WWS spectrum, due to the mostly unexplained crises of hypoglycemia that frequently occur in individuals affected by this syndrome. Importantly, severe hypoglycemic crises may in turn likely contribute to seizure susceptibility in WWS patients.

## Concluding Remarks

The main limitations of this report are the ones expectedly pertaining to a single case description being WWS an ultra-rare genetic condition. In addition, a potential impact of the sequelae of post-natal hypoxic-ischemic encephalopathy could have at least partially contributed to the clinical features of our patient. Of interest, our patient's presentation is consistent with several typical WWS-related clinical features but also indicates a novel potential pathophysiological mechanism explaining the severe hypoglycemic events (and possibly related seizures) sometimes observed in the context of this syndrome. Also, we performed a detailed investigation of the muscle biopsy phenotype of our WWS case, highlighting an abnormal NMJ morphology which potentially contributed to the neurological spectrum and the muscle weakness peculiarly associated with WWS.

## Data Availability Statement

The datasets presented in this article are not readily available due to ethical and privacy restrictions. Requests to access the datasets should be directed to the corresponding author.

## Author Contributions

GP wrote the manuscript under the mentorship of VS. Gd'A, CF, and PS acquired and interpreted the clinical data and aided in the diagnosis. FZ interpreted the genetic data and provided commentary. DT interpreted the radiologic images and provided commentary. MI and SB contributed to the genetic analysis of the family and revision of the manuscript. EA, AR, and PB acquired the clinical data. MM, EG, and CM interpreted the data and supervised the formulation of the case report. All authors contributed to the article and approved the submitted version.

## Conflict of Interest

The authors declare that the research was conducted in the absence of any commercial or financial relationships that could be construed as a potential conflict of interest.
